# LINC00355:8 promotes cell proliferation and migration with invasion *via* the MiR-6777-3p/Wnt10b axis in Hepatocellular Carcinoma

**DOI:** 10.7150/jca.43831

**Published:** 2020-07-25

**Authors:** Fangyuan Zhou, Yiming Lei, Xuan Xu, Haoxiong Zhou, Huiling Liu, Jie Jiang, Yidong Yang, Bin Wu

**Affiliations:** Department of Gastroenterology, the Third Affiliated Hospital of Sun Yat-Sen University, Guangzhou, China.

**Keywords:** Hepatocellular carcinoma, LINC00355:8, miR-6777-3p, Wnt10b

## Abstract

**Background:** Recent studies have reported that various long non-coding RNAs (lncRNAs) promote hepatocellular carcinoma (HCC) progression, and our previous study indicated that lncRNA LINC00355:8 is overexpressed in HCC. However, the role of LINC00355:8 in HCC is unclear. The primary aim of this study was to explore the biological role of LINC00355:8 in HCC.

**Methods:** Microarray analysis was performed to explore the aberrantly expressed lncRNAs in HCC compared with precancerous tissues. Real-time PCR and *in situ* hybridization were used to investigate the expression of LINC00355:8 in HCC tissues. CCK8, EdU, colony formation, wound healing and transwell assays were performed to analyse cell proliferation, migration and invasion. A xenograft tumour model was established to analyse the effect of LINC00355:8 on tumour growth *in vivo*. Luciferase assays were utilized to explore the binding sites between miR-6777-3p and other genes, such as LINC00355:8 and *Wnt10b*. After cell transfection, the protein expression levels of Wnt10b, β-catenin, E-cadherin, N-cadherin, c-Myc and Snail were determined by Western blotting.

**Results:** The present study revealed that LINC00355:8 was significantly upregulated in HCC, promoted HCC cell proliferation, migration and invasion *in vitro* and enhanced tumour growth *in vivo*. LINC00355:8 regulated miR-6777-3p expression by acting as a ceRNA, and the expression of Wnt10b was negatively modulated by miR-6777-3p. Moreover, LINC00355:8 could activate the Wnt/β-catenin signalling pathway and promote EMT progression by inhibiting the miR-6777-3p/Wnt10b interaction in HCC.

**Conclusion:** Our findings indicate that LINC00355:8 activates Wnt10b and promotes HCC progression via the suppression of miR-6777-3p, which may provide novel therapeutic targets for HCC.

## Introduction

Hepatocellular carcinoma (HCC) is the fifth most common cancer worldwide 1. The morbidity and mortality of HCC have increased significantly in recent years 2-3. However, the exact mechanism for HCC progression is still unclear. It is urgent to explore the molecular mechanism and identify novel therapeutic targets for HCC.

Protein coding genes account for only approximately 2% of the human genome. More than 97% of RNA transcripts lack protein-coding ability and are called non-coding RNAs. Of these, molecules with transcript lengths greater than 200 nucleotides are called long non-coding RNAs (lncRNAs), while microRNAs (miRNA) are 18-22 nucleotides in length 4. The distribution of lncRNAs is in both the nucleus and cytoplasm 5. LncRNAs are involved in numerous biological processes, such as cell proliferation, cell cycle progression and apoptosis 6-7. Current studies have demonstrated that aberrant expression levels of lncRNAs in tumours, including HCC, and the abnormal expression of lncRNAs may be a significant factor in tumorigenesis 8. LncRNAs play important roles in tumour growth, invasion and metastasis. For example, lncRNA-HULC (highly upregulated in HCC) and lncRNA-HEIH (high expression in HCC) have been determined to be significant factors in hepatocarcinogenesis 9-10. LINC00355:8, which was previously called Lnc-PCDH9-13:1, is located on chromosome 13, and our previous study found that the expression of LINC00355:8 is increased in HCC tissues 11. To date, the functions of LINC00355:8 in cancer, including HCC, are undetermined. miRNAs are also involved in numerous critical biological processes. Investigations have found that lncRNAs act as competitors for shared miRNA binding, termed competing endogenous RNAs (ceRNAs). Therefore, lncRNAs have the ability to regulate the expression levels of target miRNAs 12. Recent studies have revealed that some lncRNAs influence hepatocarcinogenesis by acting as ceRNAs 13. MiR-6777-3p is located on chromosome 17, the biological function of miR-6777-3p is still unclear, and the role of miR-6777-3p in HCC remains unknown. The relationship between LINC00355:8 and its putative target miR-6777-3p is not well explored.

Wnt10b is a member of the Wnt family, which plays an important role in the Wnt signalling pathway. Wnt10b is upregulated in basal cell carcinoma and skin squamous cell carcinomas to enhance tumour growth via the Wnt signalling pathway 14. However, the role of Wnt10b in HCC is unclear. Whether Wnt10b is modulated by miR-6777-3p in HCC also remains unclear.

In this study, we aimed to explore the expression level and functions of LINC00355:8 in HCC. We found that LINC00355:8 was overexpressed in HCC and promoted the progression of HCC. In addition, LINC00355:8 enhanced the cell proliferation, migration and invasion ability of HCC by activating Wnt10b via the suppression of miR-6777-3p.

## Materials and Methods

### Tissue simples

HCC samples and precancerous tissues were collected from 10 HCC patients during surgery performed at the Third Affiliated Hospital of Sun Yat-Sen University from April 2011 to August 2016. All samples were confirmed by histopathology. Patient received any chemotherapy and radiotherapy before the surgery, patient with other cancers, or patient with benign liver tumours was excluded. The clinical features of the patients are shown in Table S1. The samples were stored at -80°C. The Ethics Committee of the Third Affiliated Hospital of Sun Yat-Sen University approved the study protocol, and written informed consent was obtained from all participants.

### Microarray analysis

Total RNA was extracted from 3 HCC samples and paired normal liver tissues using TRIzol reagent (Invitrogen, Carlsbad, CA, USA). NanoDrop 2000 (Thermo Fisher Scientific, Waltham, MA, USA) was used to quantify the concentration and purity of total RNA, and standard denaturing agarose gel electrophoresis was utilized to determine RNA integrity. The microarray analysis was performed by Agilent Technologies (Agilent Technologies, Santa Clara, CA, USA). Total RNA was transcribed to fluorescent cRNA using an Agilent Quick Amp Labeling Kit. Then, the fluorescent cRNA was hybridized onto the Human LncRNA Array (8 × 60 K, Arraystar), which was scanned by an Agilent Scanner G2505C. GeneSpring software V12.0 (Agilent, USA) was used for data summarization and quantile normalization. The differential expression of lncRNAs between the two groups with statistical significance was measured by Volcano Plot filtering. The lncRNAs with fold change >2 and *P*<0.01 were selected for subsequent analysis. The expression pattern of lncRNAs was analysed by hierarchical clustering.

### Cell culture

The human liver cell line LO2 and the HCC cell lines HepG2, HepG2.2.15 and Huh7 were purchased from the Institute of Cell Biology (Chinese Academy of Science, Shanghai, China). LO2 cells were cultured in 1640 medium (Gibco, Rockville, USA) with 10% foetal bovine serum (FBS, Gibco, Rockville, USA), and HepG2, HepG2.2.15 and Huh7 cells were cultured in Dulbecco's modified Eagle's medium (DMEM, Gibco, Rockville, USA) with 10% foetal bovine serum (FBS, Gibco, Rockville, USA). All cells were maintained in a 37°C, 5% CO2 incubator.

### Cell Counting Kit-8 (CCK8), 5-ethynyl-2'-deoxyuridine (EdU) incorporation and colony formation assays

For the CCK8 assay, LO2, HepG2, HepG2.2.15 and Huh7 cells were seeded in 96-well plates at a density of 1 × 10^4^ cells per well after transfection. The CCK8 kit (Dojindo Laboratories, Japan) was used to examine cell proliferation. The optical density (OD) was read at 450 nm for 24 h, 48 h, 72 h and 96 h post seeding.

For the EdU assay, LO2, HepG2, HepG2.2.15 and Huh7 cells were seeded in 24-well plates at a density of 1 × 10^5^ cells per well after transfection and examined by an EdU kit (RiboBio, Guangzhou, China). The cells were imaged with a fluorescence microscope (Leica, Heidelberg, Germany).

For the colony formation assay, LO2, HepG2, HepG2.2.15 and Huh7 cells were seeded in 6-well plates after transfection (1 × 10^3^ cells/well). The cells were cultured at 37°C and 5% CO2 for two weeks. Finally, the cells were fixed with 4% paraformaldehyde for 30 min, stained with crystal violet and quantified with a microscope (Leica, Heidelberg, Germany).

CCK8 and EdU assays were performed according to the manufacturer's protocols.

### Cell migration assay

For the wound healing assay, transfected LO2, HepG2, HepG2.2.15 and Huh7 cells were seeded into 6-well plates (1 × 10^6^ cells/well) and grown to 90% confluence. A 100 μl pipette tip was used to scratch the cell monolayers. Then, the cells were washed with PBS three times gently and cultured in FBS-free medium for an additional incubation. Images were taken at 0 h and 24 h with an inverted microscope (Leica, Heidelberg, Germany). The results were analysed with ImageJ software (National Institutes of Health, Bethesda, USA).

For the cell Transwell assay, 200 μl (5 × 10^4^ cells) transfected LO2, HepG2, HepG2.2.15 and Huh7 cells were seeded into the upper chambers of 24-well Boyden chambers with an 8 μm pore size (BD Bioscience, CA, USA) in serum-free medium, and DMEM containing 20% FBS was added to the bottom chamber. After 24 h of incubation, the cells were fixed with 4% paraformaldehyde, dyed with crystal violet and counted with a microscope (Leica, Heidelberg, Germany).

### Cell invasion assay

For the cell invasion assay, 200 μl (1 × 10^5^ cells) transfected LO2, HepG2, HepG2.2.15 and Huh7 cells were seeded into the upper chambers of Boyden chambers coated with Matrigel (BD Bioscience, CA, USA) in serum-free medium, and DMEM containing 20% FBS was added to the bottom chamber. After 48 h of incubation, the cells were fixed with 4% paraformaldehyde, dyed with crystal violet and counted with a microscope (Leica, Heidelberg, Germany).

### Cell transfection and stable cell line construction

The LINC00355:8 siRNA, negative control siRNA, miR-6777-3p mimics, mimic NC, miR-6777-3p inhibitor and inhibitor NC were synthesized by RiboBio (Guangzhou, China). The Lv-LINC00355:8 and Lv-con viruses were synthesized by Gene (Shanghai, China). Cell transfection was carried out using Lipofectamine 3000 (Invitrogen, Carlsbad, CA, USA) according to the manufacturer's protocol. After 48 h, cells were collected for further experiments. Cells stably expressing LINC00355:8 were selected in DMEM containing puromycin (2 µg/ml) for 2 weeks.

### Quantitative real-time PCR (qRT-PCR)

Total RNA was extracted from tissues and cells using TRIzol reagent (Invitrogen, CA, USA) according to the manufacturer's instructions. Total cDNA was synthesized by ReverTra Ace qPCR RT Kit (Toyobo, Japan) for LINC00355:8 detection and miDETECT miRNA (RiboBio, Guangzhou, China) for miR-6777-3p detection according to the manufacturer's protocol. The expression level of LINC00355:8 was quantified by SYBR Green Master Mix (Finnzymes, Espoo, Finland), and the level of miR-6777-3p was examined by a miDETECT A Track miRNA qRT-PCR Starter Kit (RiboBio, Guangzhou, China) on an HT7500 system (Applied Biosystems). The LINC00355:8 expression level was normalized to the *β-actin* level, while the miR-6777-3p expression level was normalized to the *U6* level. The primer sequences were synthesized by RiboBio (Guangzhou, China).

### *In situ* hybridization

The digoxin-labelled *in situ* hybridization (ISH) probe for detecting LINC00355:8 was synthesized by Exiqon (Shanghai, China). The ISH assay was performed using an ISH Kit (Boster Company, Wuhan, China). The sequence of the probe was 5'-AGTTGTTATCCACATTCACATT-3'.

### Western blotting

The cells were lysed by cell lysis buffer (Beyotime, Shanghai, China), and the protein concentration was measured by the BCA Protein Quantification Kit (Beyotime, Shanghai, China). Equal amounts of protein were separated by SDS-polyacrylamide gel electrophoresis and transferred to nitrocellulose membranes. After blocking in 5% non-fat milk for 2 h at room temperature, the membranes were incubated with specific primary antibodies at 4°C overnight. Next, the membranes were washed with TBST three times and incubated with the secondary antibodies for 2 h at room temperature. The protein bands were detected by ECL Western blotting detection reagents (Amersham Pharmacia Biotech, Piscataway, NJ, USA) and analysed with ImageJ software (National Institutes of Health, Bethesda, USA). The primary antibodies used in this study were against β-catenin (1:1000, Cell Signaling Technology, Danvers, MA, USA, 8480), N-cadherin (1:1000, Cell Signaling Technology, 14215), E-cadherin (1:1000, Cell Signaling Technology, 14472), Snail (1:1000, Cell Signaling Technology, 3879), β-actin (1:5000, Abcam, Cambridge, MA, USA, ab179467), c-Myc (1:1000, Abcam, Cambridge, MA, USA, ab32072), and Wnt10b (1:1000, ABclonal Biotechnology Co., Ltd., A16717).

### Luciferase reporter assay

For the luciferase reporter assay, 293T cells were seeded in 24-well plates and cultured overnight before transfection. Then, the wild-type (WT) or mutated (MUT) LINC00355:8 or Wnt10b 3'-UTR was cotransfected with Renilla luciferase vector (Gene, Shanghai, China) and miR-6777-3p mimics or mimic-NC (RiboBio, Guangzhou, China) by Lipofectamine 3000 (Invitrogen, CA, USA). After 48 h, cells were collected, and the luciferase activity was measured by the Dual-Luciferase Reporter Assay System (Promega, Madison, USA). The luciferase activity of Renilla was used for normalization.

### Xenograft tumour model

Five-week-old male BALB/c athymic nude mice were purchased from Beijing Vital River Laboratory Animal Technology Co., Ltd. (Beijing, China). The mice were housed in microisolator cages under a 12/12-h light/dark cycle (lights on at 8:00 a.m.) with food and water* ad libitum*. HepG2 cells stably transfected with Lv-LINC00355:8 or Lv-con (1 × 10^7^) cells were injected subcutaneously into the right flanks of nude mice. The tumour volume was measured every 4 days using the formula (length × width × width)/2. Three weeks later, the nude mice were sacrificed, and the tumours were weighed. The animal experiments were approved by the Animal Ethics Committee of the Third Affiliated Hospital of Sun Yat-Sen University.

### Statistics analysis

All data were analysed by SPSS 19.0 software (SPSS, Chicago, IL, USA) and GraphPad Prism 7.0 (San Diego, CA, USA). The data are presented as the means ± standard deviation (SD). The differences between different groups were analysed by Student's *t*-test and one-way analysis of variance. *P*<0.05 was considered statistically significant.

## Results

### LINC00355:8 was upregulated in HCC

**Figure 1:**
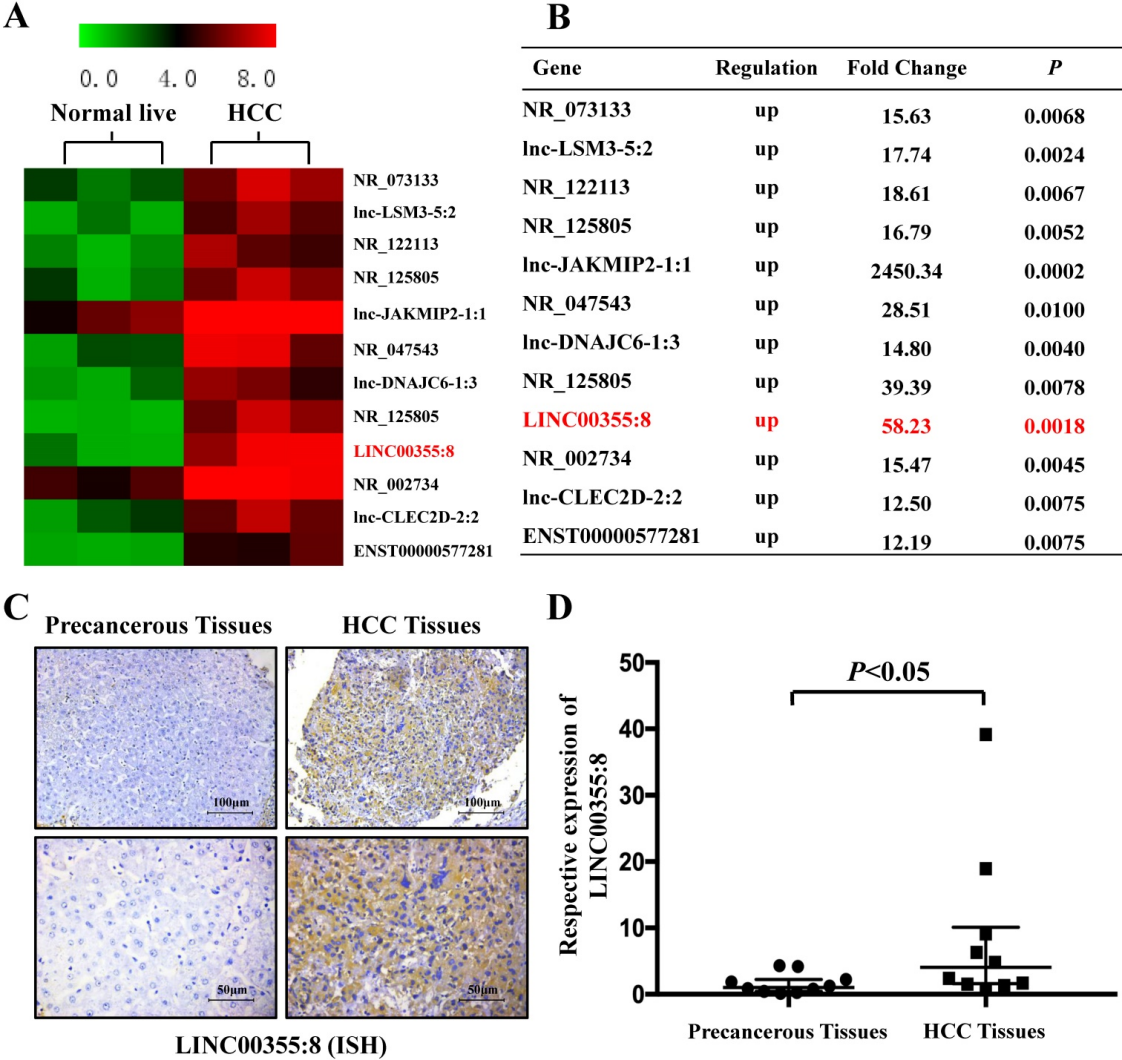
** LINC00355:8 was upregulated in HCC tissue.** (**A**) Twelve lncRNAs were upregulated between HCC tissues and paired noncancerous tissues. According to the microarray data, 12 lncRNAs were over 12-fold higher with *P*<0.01 in HCC tissues than in noncancerous tissues. Student's *t*-test was used. (**B**) Relative gene expression in HCC tissues compared with noncancerous tissues measured by qPCR. (**C**) LINC00355:8 was highly expressed in HCC tissues compared with paired noncancerous tissues, as demonstrated by ISH assay. LINC00355:8 is located in the cytoplasm of HCC tissue. (**D**) LINC00355:8 expression was significantly upregulated in HCC tissues compared with noncancerous tissues (n=10). *P*<0.05 by using one-way ANOVA.

Microarray analysis was used to identify the expression profile of lncRNAs and revealed that some lncRNAs were elevated in HCC compared with precancerous tissues. The microarray data are accessible via the Gene Expression Omnibus database (accession number GSE98269). Twelve lncRNAs were upregulated in HCC (fold change>2, *P*<0.01) (Figure 1A and B). The microarray results showed that LINC00355:8 was one of the most elevated lncRNAs in HCC. Thus, LINC00355:8 was selected for further investigation. The ISH results demonstrated that LINC00355:8 was highly expressed in HCC tissues compared with paired noncancerous tissues (Figure 1C). The ISH assay suggested that LINC00355:8 was mainly located in the cytoplasm of HCC. To confirm the ISH results, the expression of LINC00355:8 was measured by qRT-PCR in 10 HCC samples and noncancerous tissues. Consistent with the above results, LINC00355:8 was highly expressed in HCC compared with adjacent noncancerous tissues (Figure 1D). Therefore, we speculated that LINC00355:8 might be an oncogene in HCC.

### LINC00355:8 promotes HCC cell proliferation *in vitro*

**Figure 2:**
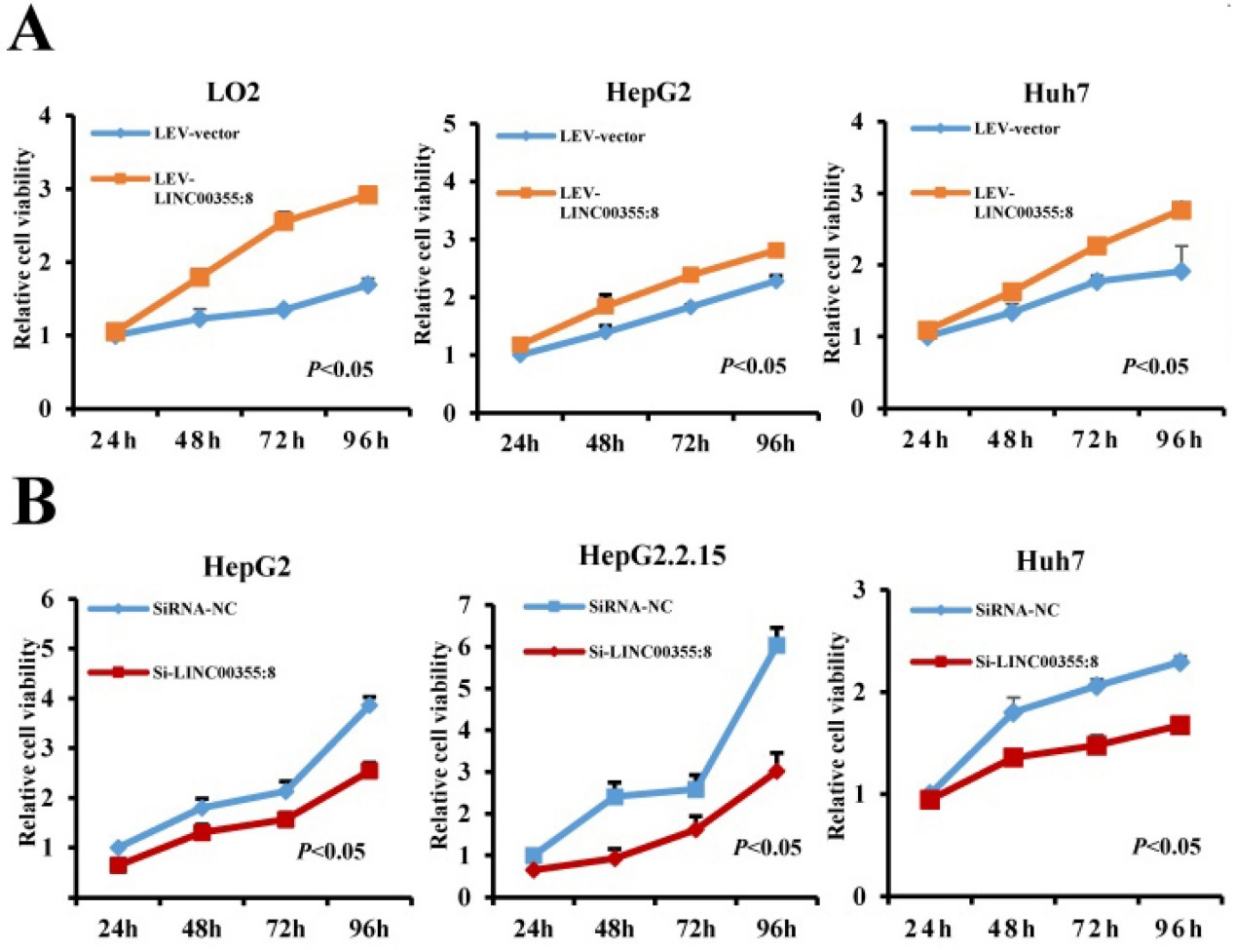

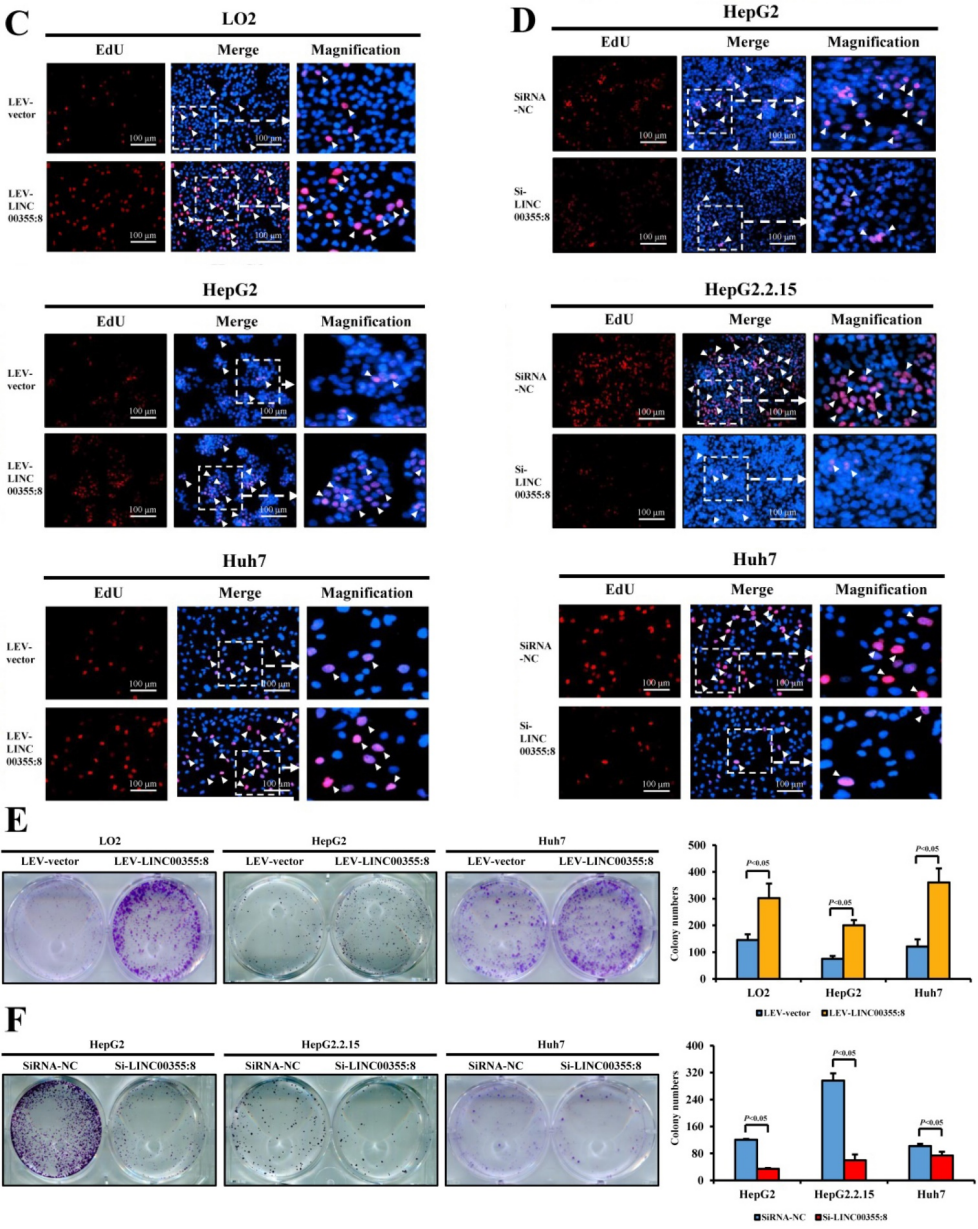
** LINC00355:8 promoted HCC cell proliferation *in vitro*.** (**A**) Overexpression of LINC00355:8 promoted cell viability as indicated by CCK8 assay. (**B**) Knockdown of LINC00355:8 inhibited cell viability as demonstrated by CCK8 assay. (**C**) The percentage of EdU-positive cells was higher in LINC00355:8-overexpressing HCC cells. (**D**) The percentage of EdU-positive cells was lower in LINC00355:8 knockdown HCC cells. (**E**) More colonies were found in LINC00355:8-overexpressing cells. (**F**) Fewer colonies were found in LINC00355:8-silenced HCC cells. Error bars represent the mean ± SD of at least three experiments. *P*<0.05 by using Student's *t*-test.

To investigate the role of LINC00355:8 in HCC, lentiviruses or siRNA were used to overexpress or knock down LINC00355:8 in cells. The transfection efficiencies were measured by qRT-PCR as shown in Figure S1. CCK8 assays, EdU assays and colony formation assays were performed to elucidate the effect of LINC00355:8 on cell proliferation. The CCK8 assay revealed that overexpression of LINC00355:8 enhanced cell activity in LO2, HepG2 and Huh7 cells, whereas knockdown of LINC00355:8 suppressed the proliferation HCC cell lines (Figure 2A and B). Similar results were obtained from the EdU assay and colony formation assay. In the EdU assay, the number of EdU-positive cells in the Lev-LINC00355:8-transfected group was higher than that in the Lev-con-transfected group (Figure 2C). In contrast, LINC00355:8 silencing reduced the number of EdU-positive cells (Figure 2D). In addition, more colonies were found in Lev-LINC00355:8-transfected cells than in Lev-con-transfected cells in the colony formation assay (Figure 2E), while LINC00355:8 knockdown inhibited colony formation (Figure 2F). The above data indicated that LINC00355:8 promoted cell proliferation in LO2, HepG2, HepG2.2.15 and Huh7 cells.

### LINC00355:8 enhanced HCC cell migration and invasion *in vitro*

**Figure 3:**
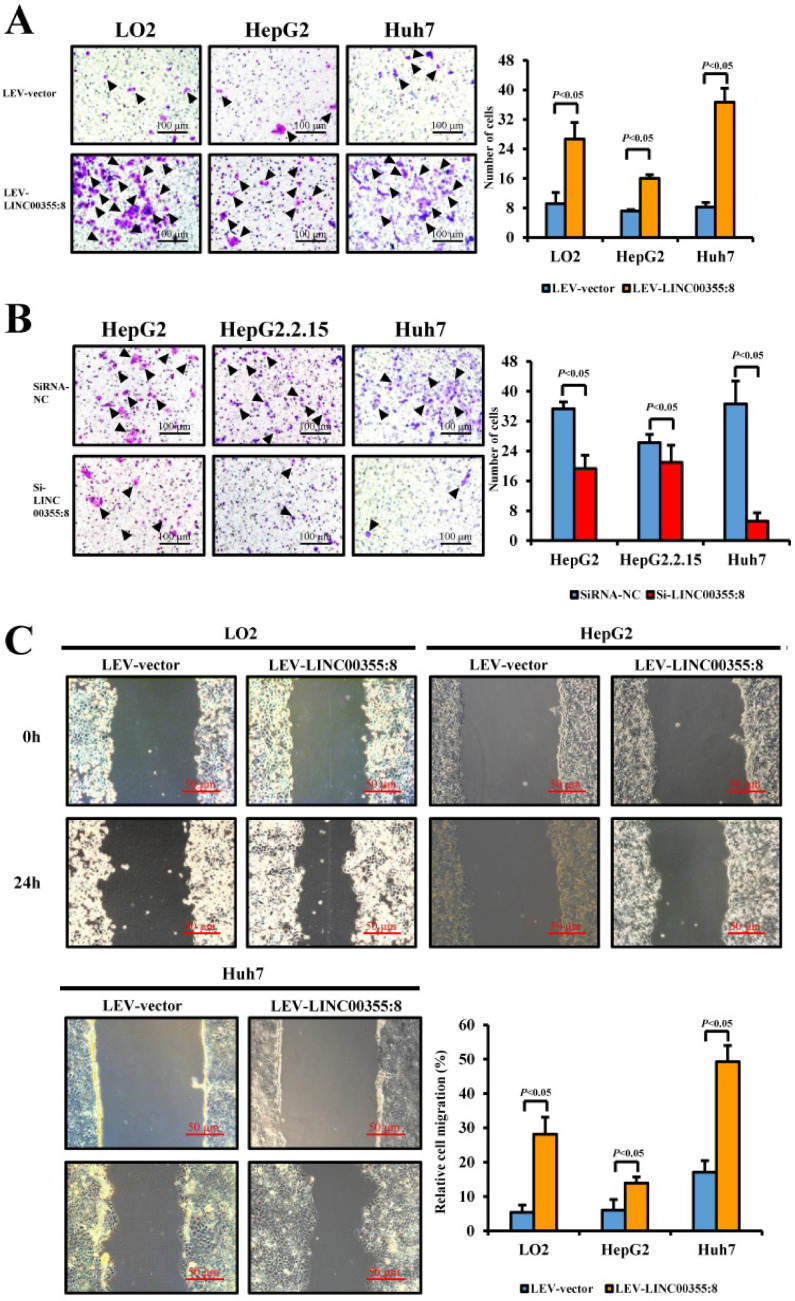

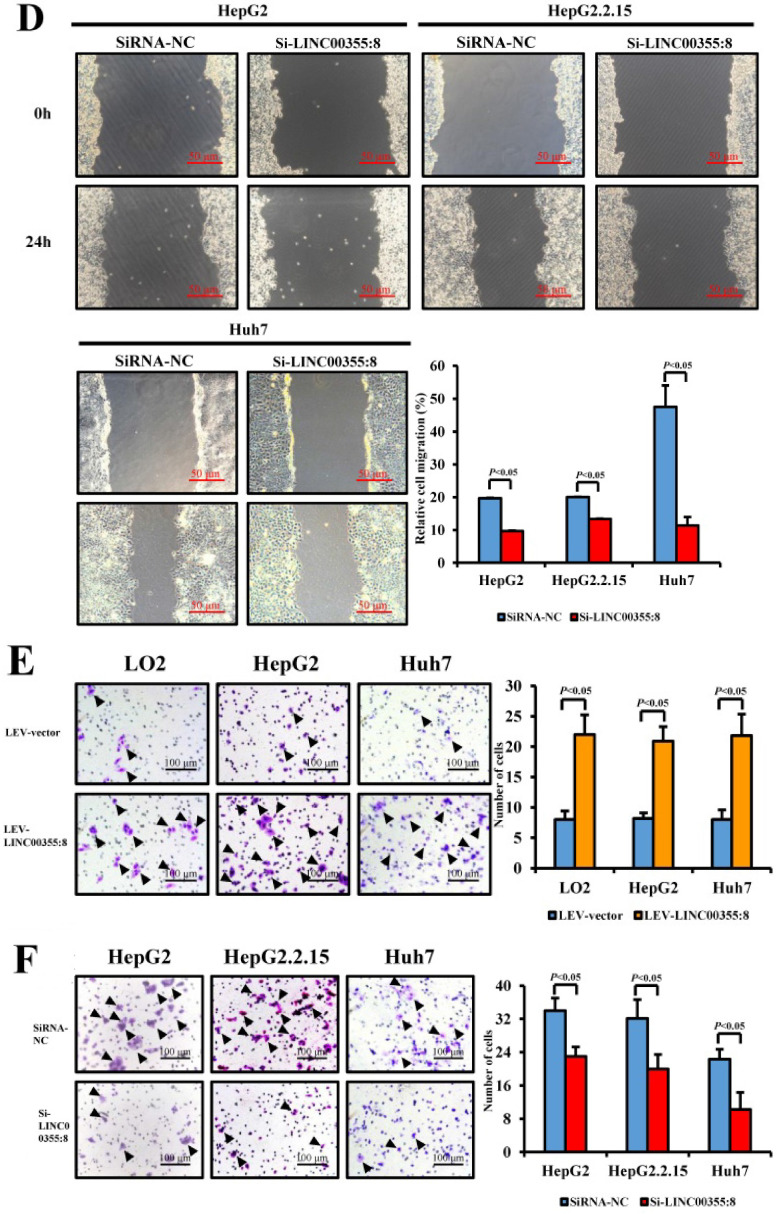
** LINC00355:8 promoted HCC cell migration and invasion *in vitro*.** (**A**) Overexpression of LINC00355:8 increased HCC cell migration ability as determined by transwell assay. (**B**) Suppression of LINC00355:8 inhibited HCC cell migration ability as determined by transwell assay. (**C**) Overexpression of LINC00355:8 promoted HCC cell migration ability as determined by wound healing assay. (**D**) Suppression of LINC00355:8 decreased HCC cell migration ability as determined by wound healing assay. (**E**) Overexpression of LINC00355:8 increased HCC cell invasion as determined by transwell assay with Matrigel. (**F**) Suppression of LINC00355:8 decreased HCC cell invasion as determined by transwell assay with Matrigel. Error bars represent the mean ± SD of at least three experiments. *P*<0.05 by using Student's *t*-test.

The relationship between LINC00355:8 expression levels and HCC malignancy prompted us to investigate whether LINC00355:8 could affect HCC cell migration and invasion. Transwell assays without Matrigel and wound healing assays were used to determine the cell migration ability, and Transwell assays with Matrigel were performed to measure the cell invasive ability. The results of the Transwell assay without Matrigel showed that LINC00355:8 overexpression facilitated the migration of LO2, HepG2 and Huh7 cells (Figure 3A). Wound healing assays demonstrated that overexpression of LINC00355:8 enhanced the mobility of LO2, HepG2 and Huh7 cells compared with control cells (Figure 3C). Conversely, a significant decrease in cell migration ability was observed in LINC00355:8-silenced HCC cells via the two above assays (Figure 3B and D). The Transwell assay with Matrigel showed that overexpression of LINC00355:8 increased the number of invaded cells in LO2, HepG2 and Huh7 cells. The opposite results were observed in LINC00355:8 knockdown HCC cells (Figure 3E and F). These results collectively suggested that LINC00355:8 enhanced HCC cell migration and invasion *in vitro*.

### LINC00355:8 promotes HCC growth *in vivo*

**Figure 4:**
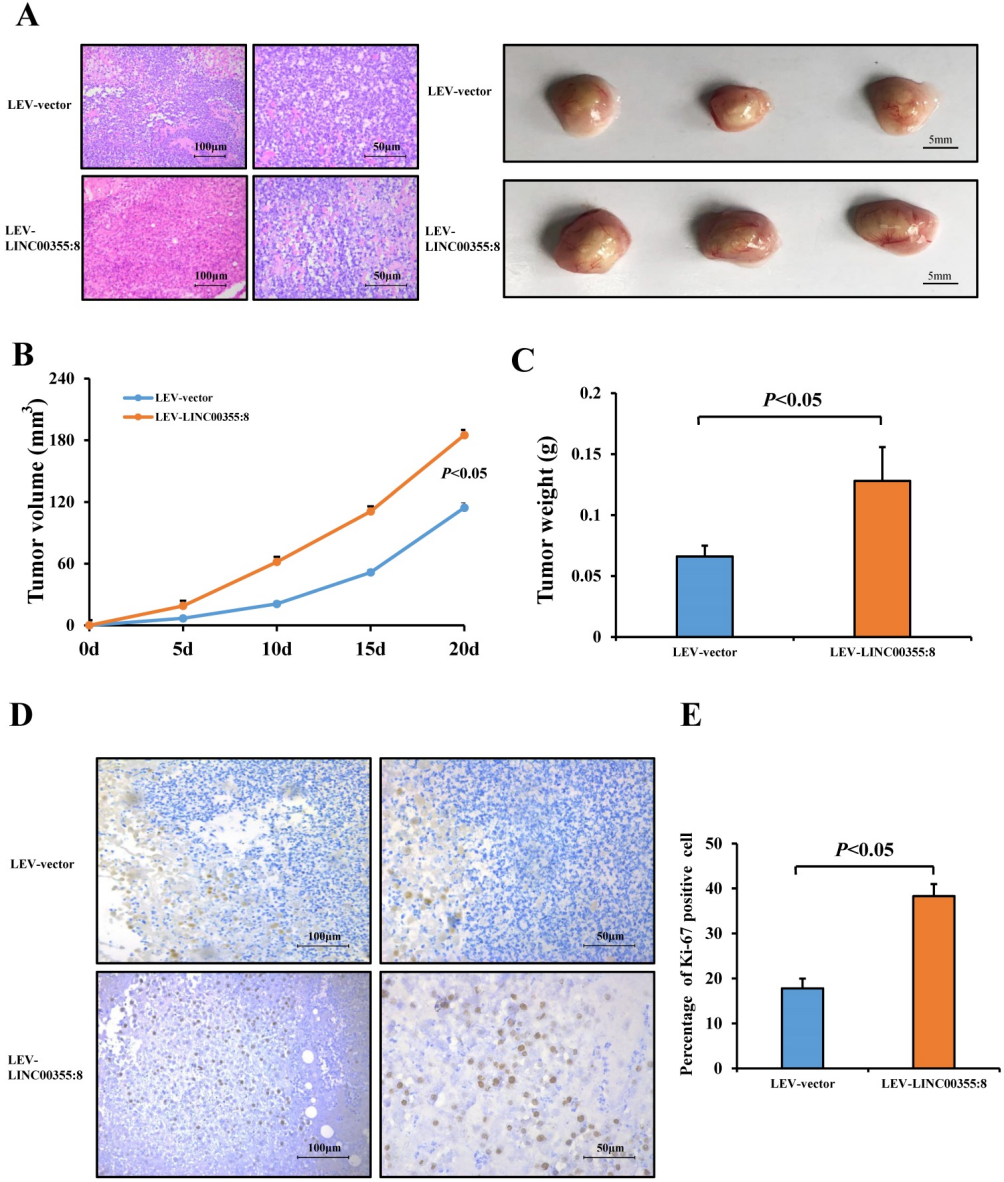
** LINC00355:8 promoted HCC growth *in vivo*.** (**A**) Representative H&E staining images of tumour nodes at 20 days in the LINC00355:8 overexpression and control groups. (**B-C**) Tumours of the LINC00355:8 overexpression group had a larger volume and heavier weight than those of the control group. (**D-E**) Overexpression of LINC00355:8 increased the percentage of Ki-67-positive cells in tumours compared with those of the control group. Error bars represent the mean ± SD of at least three experiments. *P*<0.05 by using Student's *t*-test.

Then, a subcutaneous xenograft tumour model was generated to elucidate the role of LINC00355:8* in vivo*. The results showed that LINC00355:8 overexpression enhanced HCC cell-derived tumour growth (Figure 4A), as the volume and weight of tumour nodes were increased in mice injected with LINC00355:8-overexpressing cells compared with the control group (Figure 4B and C). Furthermore, Ki-67 expression was measured in tumours in both the LINC00355:8 overexpression and control groups to quantitatively determine the proliferation ability of the xenograft tumours. Immunohistochemistry for Ki-67 showed that overexpression of LINC00355:8 increased the percentage of Ki-67-positive cells in tumours (Figure 4D and E). These findings suggested that LINC00355:8 promoted HCC growth *in vivo*.

### LINC00355:8 regulates miR-6777-3p by acting as a ceRNA

**Figure 5:**
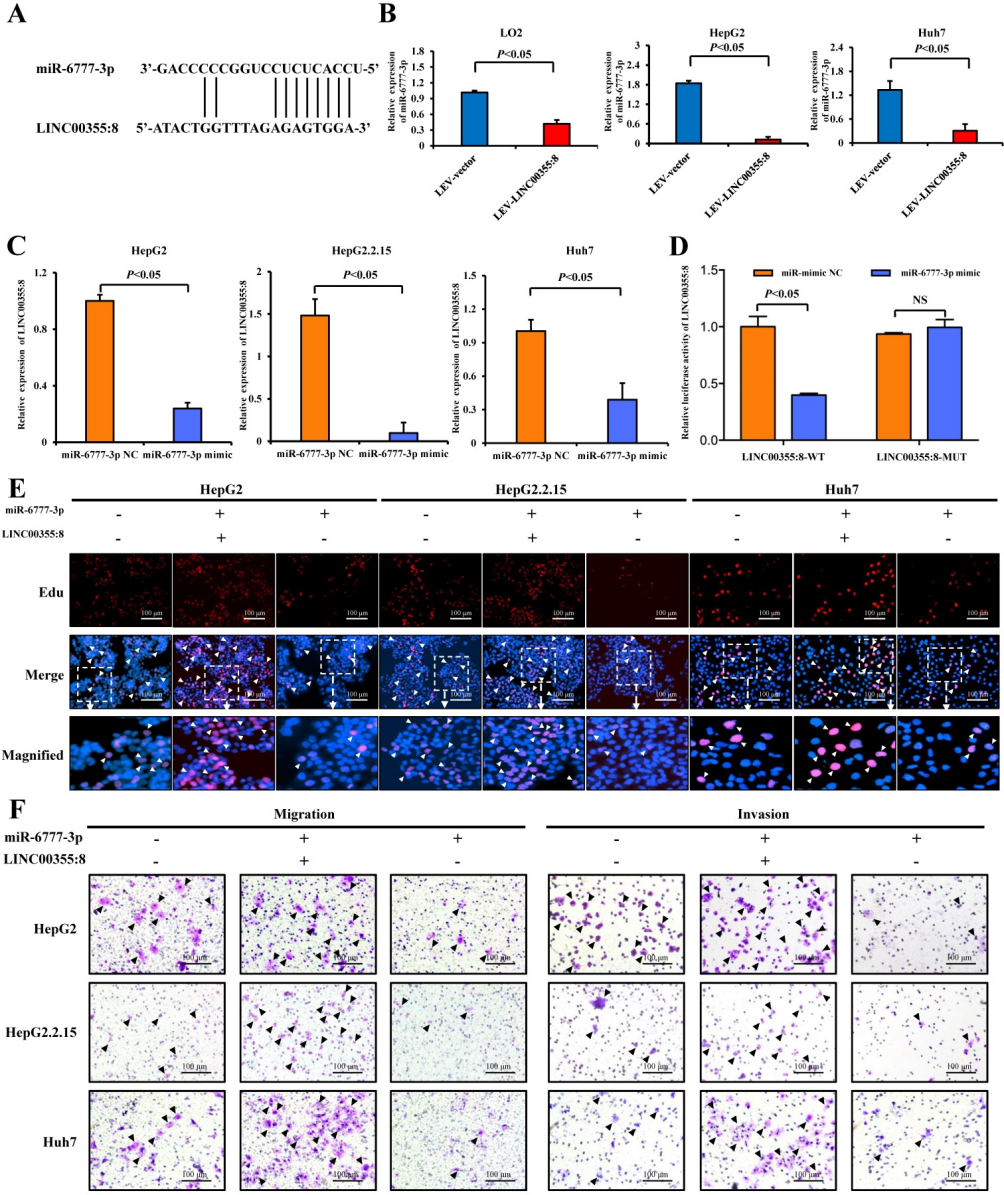
** LINC00355:8, functioning as a ceRNA, regulated miR-6777-3p.** (**A**) The predicted binding site between LINC00355:8 and miR-6777-3p. (**B**) Expression levels of miR-6777-3p in LO2, HepG2 and Huh7 cells transfected with Lev-LINC00355:8 or Lev-NC. (**C**) Expression levels of LINC00355:8 in HepG2, HepG2.2.15 and Huh7 cells transfected with miR-6777-3p mimic or miR-mimic NC. (**D**) 293T cells were cotransfected with miR-6777-3p mimic or miR-mimic NC and the luciferase reporter plasmid containing LINC00355:8-WT or LINC00355:8-MUT. The luciferase activity was normalized to Renilla luciferase activity. (**E-F**) MiR-6777-3p suppressed the promoting effects of LINC00355:8 on the proliferation, migration and invasion of HepG2, HepG2.2.15 and Huh7 cells. Error bars represent the mean ± SD of at least three experiments. *P*<0.05 by using Student's *t*-test.

A growing number of studies have revealed that lncRNAs can regulate cell biological behaviours as ceRNAs or molecular sponges of miRNAs involved in cancer progression 15. To investigate whether LINC00355:8 plays a similar role in regulating miRNAs, the online software programme lncRNASNP2 (http://bioinfo.life.hust.edu.cn/lncRNASNP) was utilized to predict the potential interaction between LINC00355:8 and target miRNAs. Of the potential miRNAs, only the expression level of miR-6777-3p was decreased by the overexpression of LINC00355:8 (Figure 5A and B). Furthermore, the expression of LINC00355:8 was negatively regulated by miR-6777-3p (Figure 5C). The predicted wild-type miR-6777-3p binding site in LINC00355:8 and mutated miR-6777-3p binding site in LINC00355:8 were cloned into the luciferase reporter plasmid to construct vectors termed LINC00355:8-WT and LINC00355:8-MUT, respectively. Then, a luciferase assay was performed to investigate whether LINC00355:8 bound to miR-6777-3p. The results revealed that overexpression of miR-6777-3p significantly decreased the luciferase activity of LINC00355:8-WT but not that of LINC00355:8-MUT (Figure 5D). These results suggested that miR-6777-3p directly bound to LINC00355:8.

Further investigations were performed to explore whether LINC00355:8 played a role in cell proliferation, migration and invasion through miR-6777-3p. As shown in the EdU assay, overexpression of miR-6777-3p suppressed the promoting effect of LINC00355:8 on the proliferation of HepG2, HepG2.2.15 and Huh7 cells (Figure 5E). Transwell assay results suggested that overexpression of miR-6777-3p abolished the promoting effect of LINC00355:8 on the migration of HCC cells (Figure 5F). Accordingly, miR-6777-3p inhibited the regulatory effect of LINC00355:8 on cell invasive ability (Figure 5F). All these results demonstrated that LINC00355:8 exerted its biological functions as a ceRNA of miR-6777-3p.

### LINC00355:8 regulates Wnt10b through miR-6777-3p

**Figure 6:**
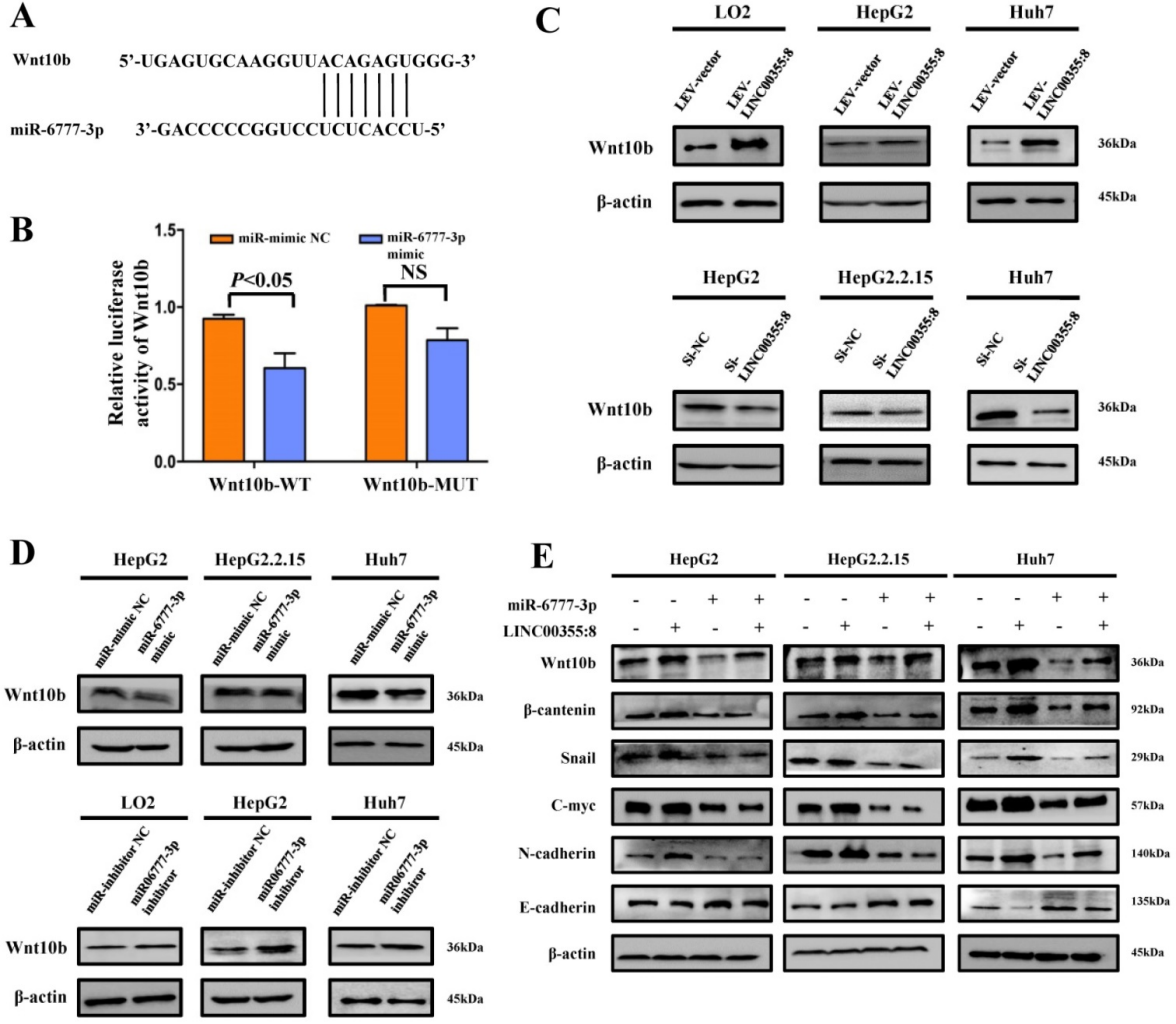
** LINC00355:8 regulated Wnt10b through inhibition of miR-6777-3p.** (**A**) The predicted binding site between miR-6777-3p and Wnt10b. (**B**) 293T cells were cotransfected with miR-6777-3p mimic or miR-mimic NC and the luciferase reporter plasmid containing Wnt10b-WT or Wnt10b-MUT. The firefly luciferase activity was normalized to Renilla luciferase activity. (**C**) The protein level of Wnt10b in LO2, HepG2, HepG2.2.15 and Huh7 cells transfected with Lev-LINC00355:8, Lev-NC, si-LINC00355:8 or si-NC. (**D**) The protein levels of Wnt10b in LO2, HepG2, HepG2.2.15 and Huh7 cells transfected with miR-6777-3p, miR-mimic NC, miR-6777-3p inhibitor or miR-inhibitor NC. (**E**) Western blot analysis of Wnt10b, β-catenin, N-cadherin, Snail, and E-cadherin protein levels in HepG2, HepG2.2.15 and Huh7 cells after transfection with Lev-NC and miR-mimic NC, Lev-LINC00355:8 and miR-mimic NC, Lev-LINC00355:8 and miR-6777-3p mimic or Lev-NC and miR-mimic NC. Error bars represent the mean ± SD of at least three experiments.* P*<0.05 by using Student's *t*-test.

**Figure 7:**
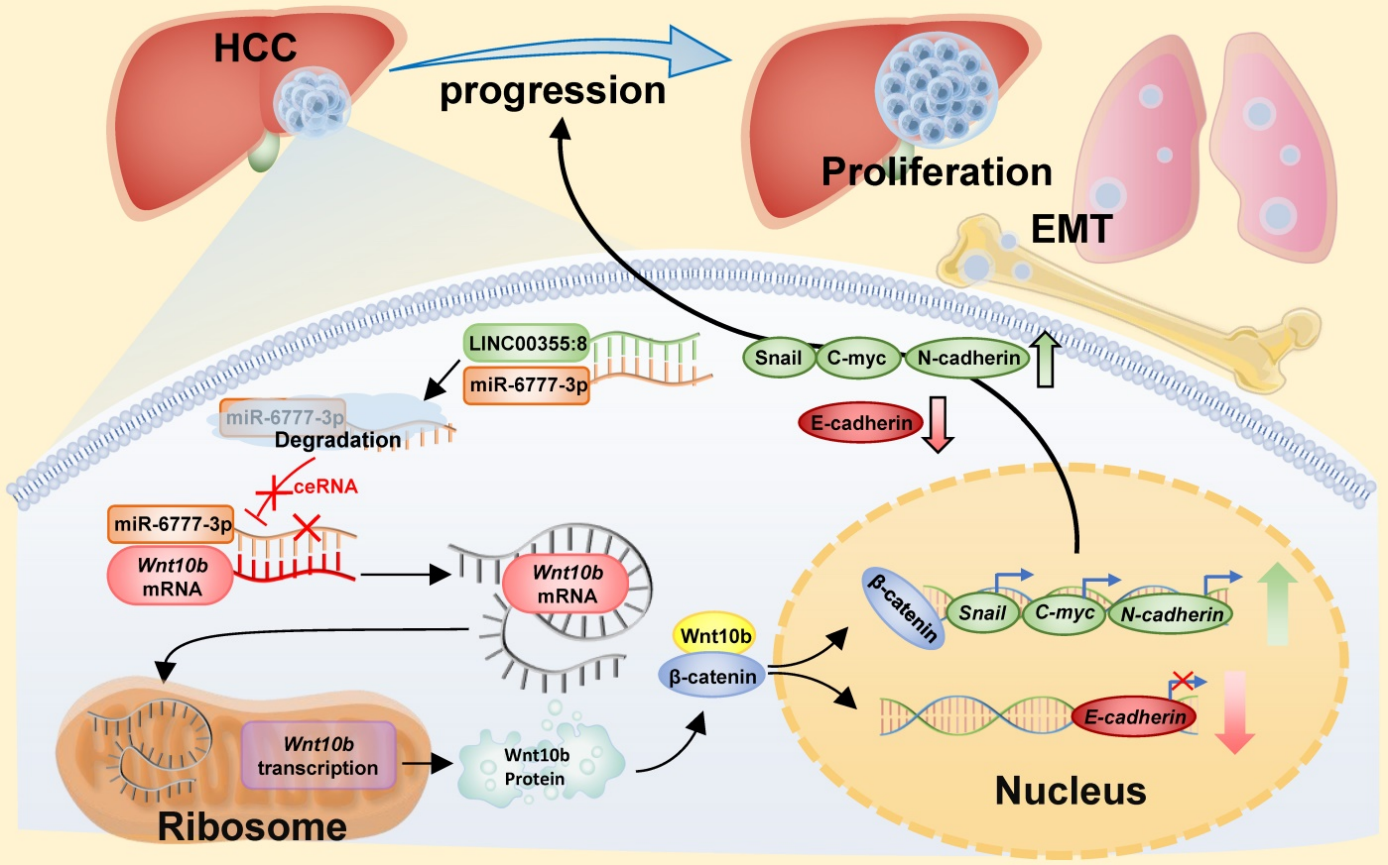
** Diagram of the mechanism of LINC00355:8 in HCC.** Overexpression of LINC00355:8 activated the Wnt/β-catenin signalling pathway via the LINC00355:8/miR-6777-3p/Wnt10b axis to regulate progression, proliferation and EMT in HCC.

The above results indicated that LINC00355:8 enhanced cell proliferation, migration and invasion through the suppression of miR-6777-3p by acting as a ceRNA. To further identify downstream target genes of miR-6777-3p participating in LINC00355:8-regulated HCC progression, the bioinformatics tool TargetScan (http://www.targetscan.org/vert_72/) was utilized, and Wnt10b, a member of the Wnt family, was predicted to be a target gene of miR-6777-3p (Figure 6A). The predicted wild-type miR-6777-3p binding site in the Wnt10b 3`UTR and mutated miR-6777-3p binding site in the Wnt10b 3'UTR were cloned into the luciferase reporter plasmid to construct vectors termed Wnt10b-WT and Wnt10b-MUT, respectively. A luciferase reporter assay showed that miR-6777-3p negatively regulated the luciferase activity of Wnt10b-WT but not Wnt10b-MUT (Figure 6B). Then, we attempted to examine whether Wnt10b was a target of miR-6777-3p in HCC cells. In LO2, HepG2 and Huh7 cells, overexpression of LINC00355:8 increased Wnt10b expression; in contrast, downregulation of LINC00355:8 decreased Wnt10b protein expression (Figure 6C). Moreover, up- or downregulated miR-6777-3p expression could decrease or increase Wnt10b protein expression, respectively (Figure 6D). To further validate the relationship between LINC00355:8, miR-6777-3p and Wnt10b, we transferred Lev-NC with mimic NC, Lev-LINC00355:8 plus mimic NC, Lev-LINC00355:8 with miR-6777-3p mimic and Lev-NC plus miR-6777-3p-mimic into HepG2, HepG2.2.15 and Huh7 cells and measured Wnt10b protein levels. The results indicated that overexpression of LINC00355:8 increased Wnt10b protein levels, while upregulation of miR-6777-3p decreased LINC00355:8-induced Wnt10b protein levels (Figure 6E). A previous study found that Wnt10b is an activator of the Wnt/β-catenin signalling pathway 16. Western blot analysis of HepG2, HepG2.2.15 and Huh7 cells revealed that LINC00355:8 not only increased the expression of β-catenin and c-Myc but also upregulated the expression of marker proteins of epithelial-mesenchymal transition (EMT), including N-cadherin and Snail, and decreased the expression of E-cadherin. Furthermore, the promoting effect of LINC00355:8 could be reversed by the miR-6777-3p mimic (Figure 6E). These data suggested that overexpression of LINC00355:8 activates the Wnt/β-catenin signalling pathway via the LINC00355:8/miR-6777-3p/Wnt10b axis to regulate the progression of EMT (Figure 7).

## Discussion

To our knowledge, our study is the first to reveal the expression level and biological function of LINC00355:8 in HCC. The present study showed that LINC00355:8 was upregulated in HCC. Moreover, LINC00355:8 promoted HCC cell proliferation, migration and invasion ability *in vitro* and enhanced tumour growth *in vivo*. Mechanistically, LINC00355:8, binding with miRNA-6777-3p as a ceRNA, regulated Wnt10b expression and promoted the progression of EMT.

Recent studies have indicated that non-coding RNAs, especially miRNAs and lncRNAs, are considered effective diagnostic biomarkers and therapeutic targets for HCC 17. An increasing number of studies have identified numerous lncRNAs associated with HCC, such as DGCR5 and lncRNA PCAT-1. These lncRNAs contribute to the regulation of the proliferation, migration and invasion of HCC cells 18-19. Our previous study showed that LINC00355:8 expression is increased in HCC compared with normal liver tissue; however, the biological function of LINC00355:8 in HCC remains unknown 11. We investigated the role of LINC00355:8 in HCC cells via functional studies in cells. As expected, LINC00355:8 enhanced HCC cell proliferation, migration and invasion *in vitro* and increased tumour growth in the nude mouse model. These data revealed that LINC00355:8 may be an oncogene that plays an important role in promoting HCC progression.

Previous studies have demonstrated a regulatory pattern between lncRNAs and miRNAs. LncRNAs, which contain miRNA binding sites, could negatively regulate the expression level of miRNAs 13. LncRNAs mainly maintain a steady expression level in the cytoplasm to function as effective miRNA sponges 20. For instance, lncRNA ANRIL inhibits HCC cell proliferation and invasion by acting as a ceRNA for miR-122-5p 21. LncRNA DSCR8 competitively binds with miR-485-5p and contributes to HCC progression 22. From the ISH assay, LINC00355:8 was located in the cytoplasm and had the potential to be a ceRNA. We wondered whether LINC00355:8 contained binding sites for miRNAs, and miR-6777-3p was selected through online prediction software for further study. The expression of miR-6777-3p was significantly decreased by LINC00355:8 overexpression. The luciferase reporter assay indicated that LINC00355:8 could bind to miR-6777-3p on the predicted binding site. Similar to other reported lncRNAs, such as lncRNA SNHG5, which acts as an endogenous sponge to bind with miR-26a-5p and decrease miRNA-induced repression of GSK3β 23, LINC00355:8 acted as a ceRNA by binding to miR-6777-3p to inhibit miR-6777-3p binding to target mRNAs. The biological function of miR-6777-3p has not yet been reported. Experiments *in vitro* revealed that miR-6777-3p inhibited the promoting effect of LINC00355:8 on the proliferation, migration and invasion of HCC cells. From these results, we revealed that miR-6777-3p might be a tumour suppressor in HCC, and the results strongly suggested that LINC00355:8 acts as a ceRNA to regulate miR-6777-3p in HCC cells.

Through bioinformatics tools, we found that Wnt10b, a member of the Wnt family, may be the target of miR-6777-3p. As a significant member of the Wnt family, Wnt10b can activate the Wnt/β-catenin signalling pathway and contribute to the biological functions of cancer cells 16. Wnt10b is considered a potential oncogene in human cancer that interacts with other Wnt family members to promote the Wnt/β-catenin signalling pathway 24-25. A previous study showed that Wnt10b silencing reduces HCC cell proliferation ability 26. However, the mechanism of Wnt10b in HCC cells remains unclear. MiRNAs can directly target mRNAs and regulate their expression at the post-transcriptional level. For instance, miR-575 negatively modulated ST7L expression by directly targeting the ST7L 3'UTR 27. In our study, the luciferase reporter assay and Western blot assay indicated that miR-6777-3p targeted Wnt10b and regulated its expression, which indicated that Wnt10b was a downstream target of miR-6777-3p. In a previous study, a similar mechanism was found. Among colorectal cancer cells, researchers have found that miR-148a suppresses cell invasion and migration by targeting wnt10b and modulating Wnt/β-catenin signalling 28. EMT is a vital process in promoting cell migration and invasion 29. A previous study illustrated that the activation of the Wnt/β-catenin pathway decreases the expression of E-cadherin and induces the EMT process 30. In our study, the results suggested that miR-6777-3p mediated LINC00355:8-induced activation of Wnt/β-catenin signalling and EMT progression by regulating Wnt10b. c-Myc is a target gene of the Wnt/β-catenin signalling pathway 31. Similar results were shown in our study, and the expression level of c-Myc had the same trend as β-catenin expression. In addition, it has been reported that lncRNAs are involved in the EMT phenotype. For example, LINC01287 promotes EMT progression to enhance HCC cell invasion via the LINC01287/miR-298/STAT3 feedback loop 32. LncRNA UCA1 promotes EMT by enhancing Wnt/β-catenin signalling in breast cancer cells 33. Thus, LINC00355:8 activated the Wnt/β-catenin signalling pathway via the miR-6777-3p/Wnt10b axis to promote EMT progression in HCC.

## Conclusions

In summary, our study revealed for the first time that LINC00355:8 is an oncogene in HCC. We further demonstrated that LINC00355:8 activated the Wnt/β-catenin signalling pathway to promote EMT progression in HCC by increasing Wnt10b expression via the suppression of miR-6777-3p. Our findings suggest that LINC00355:8 and miR-6777-3p might be novel potential therapeutic targets for HCC.

## Supplementary Material

Supplementary figure and table.

## Abbreviations

lncRNA: long non-coding RNA
HCC: hepatocellular carcinoma
microRNA: miRNA
ceRNA: competing endogenous RNA
DMEM: Dulbecco's modified Eagle's medium
CCK8: Cell Counting Kit-8
EdU: 5-ethynyl-2'-deoxyuridine
OD: optical density
qRT-PCR: quantitative real-time PCR
ISH: *in situ* hybridization
WT: wild-type
MUT: mutated
SD: standard deviation
EMT: epithelial-mesenchymal transition

## References

[1] Yang JD, Hainaut P, Gores GJ (2019). A global view of hepatocellular carcinoma: trends, risk, prevention and management. Nat Rev Gastroenterol Hepatol.

[2] Bray F, Ferlay J, Soerjomataram I (2018). Global cancer statistics 2018: GLOBOCAN estimates of incidence and mortality worldwide for 36 cancers in 185 countries. CA Cancer J Clin.

[3] Torre LA, Bray F, Siegel RL (2015). Global Cancer Statistics, 2012. CA Cancer J Clin.

[4] Carthew RW, Sontheimer EJ (2009). Origins and Mechanisms of miRNAs and siRNAs. Cell.

[5] Qiu MT, Hu JW, Yin R (2013). Long noncoding RNA: an emerging paradigm of cancer research. Tumor Biol.

[6] Lee JT (2012). Epigenetic Regulation by Long Noncoding RNAs. Science.

[7] Cech TR, Steitz JA (2014). The Noncoding RNA Revolution-Trashing Old Rules to Forge New Ones. Cell.

[8] Spizzo R, Almeida MI, Colombatti A (2012). Long non-coding RNAs and cancer: a new frontier of translational research?. Oncogene.

[9] Wang JY, Liu XF, Wu HC (2010). CREB up-regulates long non-coding RNA, HULC expression through interaction with microRNA-372 in liver cancer. Nucleic Acids Res.

[10] Yang F, Zhang L, Huo XS (2011). Long Noncoding RNA High Expression in Hepatocellular Carcinoma Facilitates Tumor Growth Through Enhancer of Zeste Homolog 2 in Humans. Hepatology.

[11] Xie ZJ, Zhou FY, Yang YD (2018). Lnc-PCDH9-13:1 Is a Hypersensitive and Specific Biomarker for Early Hepatocellular Carcinoma. EBioMedicine.

[12] Dasgupta P, Kulkarni P, Majid S (2018). MicroRNA-203 inhibits long noncoding RNA HOTAIR and regulates tumorigenesis through epithelial-to-mesenchymal transition pathway in renal cell carcinoma. Mol Cancer Ther.

[13] Karreth FA, Pandolfi PP (2013). ceRNA Cross-Talk in Cancer: When ce-bling Rivalries Go Awry. Cancer Discov.

[14] Li YH, Zhang K, Ye JX (2011). Wnt10b promotes growth of hair follicles via a canonical Wnt signalling pathway. Clin Exp Dermatol.

[15] Tay Y, Rinn J, Pandolfi PP (2014). The multilayered complexity of ceRNA crosstalk and competition. Nature.

[16] Wend P, Wend K, Krum SA (2012). The role of WNT10B in physiology and disease. Acta Physiol (Oxf).

[17] Wong CM, Tsang FH, Ng IO (2018). Non-coding RNAs in hepatocellular carcinoma: molecular functions and pathological implications. Nat Rev Gastroenterol Hepatol.

[18] Wang XL, Shi M, Xiang T (2019). Long noncoding RNA DGCR5 represses hepatocellular carcinoma progression by inactivating Wnt signaling pathway. J Cell Biochem.

[19] Zhang DY, Cao JY, Zhong QL (2017). Long noncoding RNA PCAT-1 promotes invasion and metastasis via the miR-129-5p-HMGB1 signaling pathway in hepatocellular carcinoma. Biomed Pharmacother.

[20] Liu D, Li YW, Luo G (2017). LncRNA SPRY4-IT1 sponges miR-101-3p to promote proliferation and metastasis of bladder cancer cells through up-regulating EZH2. Cancer Lett.

[21] Ma J, Li TF, Han XW (2018). Knockdown of LncRNA ANRIL suppresses cell proliferation, metastasis, and invasion via regulating miR-122-5p expression in hepatocellular carcinoma. J Cancer Res Clin Oncol.

[22] Wang YF, Sun LK, Wang L (2018). Long non-coding RNA DSCR8 acts as a molecular sponge for miR-485-5p to activate Wnt/β-catenin signal pathway in hepatocellular carcinoma. Cell Death Dis.

[23] Li YR, Guo D, Zhao Y (2018). Long non-coding RNA SNHG5 promotes human hepatocellular carcinoma progression by regulating miR-26a-5p/GSK3β signal pathway. Cell Death Dis.

[24] Chen HM, Wang YM, Xue FX (2013). Expression and the clinical significance of Wnt10a and Wnt10b in endometrial cancer are associated with the Wnt/β-catenin pathway. Oncol Rep.

[25] Wu XD, Bie QL, Zhang B (2017). Wnt10B is critical for the progression of gastric cancer. Oncol Lett.

[26] Wu GH, Fan XL, Sun L (2015). Wnt10B silencing reduces viability of HCC cells. Am J Cancer Res.

[27] Yan SY, Tang ZR, Chen K (2018). Long noncoding RNA MIR31HG inhibits hepatocellular carcinoma proliferation and metastasis by sponging microRNA-575 to modulate ST7L expression. J Exp Clin Cancer Res.

[28] Shi L, Xi JL, Xu XM (2019). MiR-148a suppressed cell invasion and migration via targeting WNT10b and T modulating β-catenin signaling in cisplatin-resistant colorectal cancer cells. Biomed Pharmacother.

[29] Xu Q, Deng F, Qin Y (2016). Long non-coding RNA regulation of epithelial- mesenchymal transition in cancer metastasis. Cell Death Dis.

[30] Clevers H, Nusse R (2012). Wnt/β-Catenin Signaling and Disease. Cell.

[31] Bahrami A, Amerizadeh F, ShahidSales S (2017). Therapeutic Potential of Targeting Wnt/β-Catenin Pathway in Treatment of Colorectal Cancer: Rational and Progress. J Cell Biochem.

[32] Mo YC, He LG, Lai ZR (2018). LINC01287/miR-298/STAT3 feedback loop regulates growth and the epithelial-to-mesenchymal transition phenotype in hepatocellular carcinoma cells. J Exp Clin Cancer Res.

[33] Xiao C, Wu CH, Hu HZ (2016). LncRNA UCA1 promotes epithelial-mesenchymal transition (EMT) of breast cancer cells via enhancing Wnt/beta-catenin signaling pathway. Eur Rev Med Pharmacol Sci.

