# Supplementary material for: LINC00355:8 promotes cell proliferation and migration with invasion via the MiR-6777-3p/Wnt10b axis in Hepatocellular Carcinoma
Source: J Cancer. 2020 Jul 25;11(19):5641–55. doi: 10.7150/jca.43831 (PMC7477441; doi:10.7150/jca.43831)
Supplement: Supplementary file 1 — Supplementary figure and table. [file jcav11p5641s1.pdf]

Table S1 Characteristics of HCC patients

Parameter	Number of patients
Age (years)	
≤40	1
40-60	3
≥60	6
Gender	
Male	7
Female	3
HBV infection	
Yes	7
No	3
Tumor size (cm)	
<5	6
≥5	4
TNM stage	
I-II	7
III-IV	3
Liver Cirrhosis	
Yes	5
No	5

Abbreviations: HBV, hepatitis B virus; TNM, tumor node metastasis.

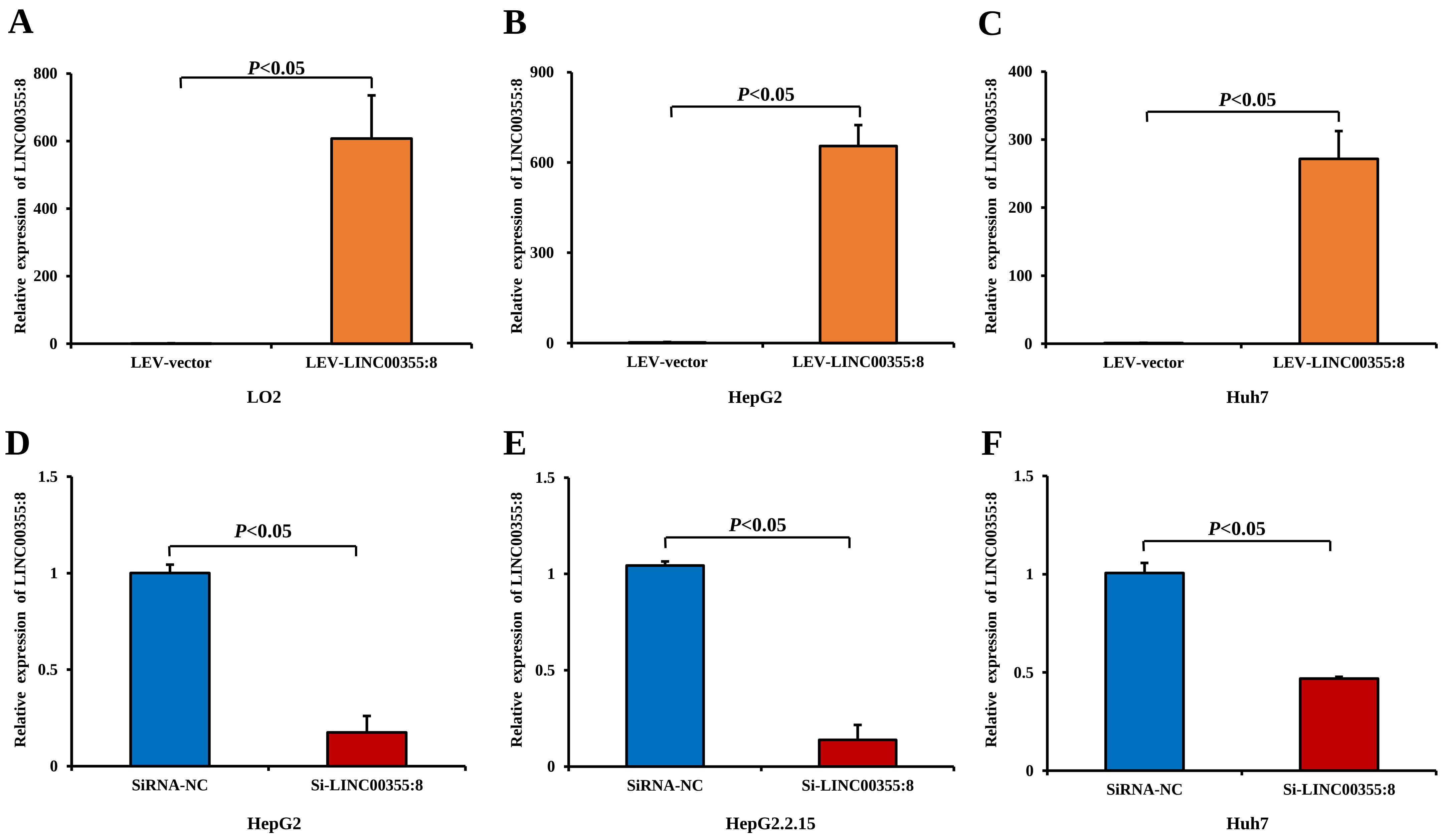

Figure S1. The transfection efficiencies were measured in LO2, HepG2, Huh7 and HepG2.2.15 cells by qRT-PCR. A-C. The expression level of LINC00355:8 is increased after transfection with LINC00355:8 lentiviruses in LO2, HepG2 and Huh7 cells. D-F. The expression level of LINC00355:8 was decreased after transfection with LINC00355:8 siRNA in HepG2 HepG2.2.15 and Huh7 cells. Error bars represent the mean ± SD of at least three experiments. $P<0.05$ by using Student's *t*-test.
